# AI and algorithmic literacy among health workers: a scoping review through a digital health literacy lens

**DOI:** 10.3389/fpubh.2026.1802392

**Published:** 2026-04-23

**Authors:** Ihoghosa Iyamu, Carly Wheelans, Devon Haag, Ian Roe, Hsiu-Ju Chang, Lorie Donelle, Ursula Ellis, Geoffrey McKee, Sofia Bartlett, Mark Gilbert

**Affiliations:** 1 School of Population and Public Health (SPPH), University of British Columbia (UBC), Vancouver, BC, Canada; 2 British Columbia Centre for Disease Control (BCCDC), Vancouver, BC, Canada; 3 School of Public Health Sciences, University of Waterloo, Waterloo, ON, Canada; 4 College of Nursing, University of South Carolina, Columbia, SC, United States; 5 Woodward Library, University of British Columbia, Vancouver, BC, Canada

**Keywords:** AI literacy, algorithmic literacy, artificial intelligence, digital health literacy, health workforce, measurement, public health, scoping review

## Abstract

**Introduction:**

Artificial intelligence (AI) and algorithmic systems influence health workers’ access, interpretation, and action on clinical and public health information, positioning them as intermediaries between algorithmically mediated outputs and patients, communities, and decision makers. This study examines how AI and algorithmic literacy are conceptualized and measured among health workers through a digital health literacy (DHL) lens.

**Methods:**

Using Arksey and O’Malley’s scoping review framework, we searched Ovid MEDLINE, Ovid Embase, Scopus, IEEE Xplore, ACM Digital Library, Europe PMC, and arXiv for English language sources published between January 2020 and May 2025. Two reviewers screened records and extracted data using a theory informed charting framework grounded in Nutbeam’s model (functional: basic understanding and use; critical: evaluation and ethics; communicative: interacting with AI systems and explaining AI-mediated information). We synthesized findings using descriptive statistics and a narrative synthesis.

**Results:**

Twelve studies published between 2021 and 2025 met inclusion criteria. Evidence was concentrated in health professions education (10/12), primarily among medical (6/12) and nursing students (2/12), with no studies exploring public health practice. Explicit, theory-grounded definitions of AI literacy were uncommon, and links to DHL were only implied. AI literacy was frequently operationalized through self-reported instruments, commonly the Artificial Intelligence Literacy Scale (AILS; 3 studies), Meta Artificial Intelligence Literacy Scale (MAILS; 2 studies) and the Scale for the Assessment of Non-Experts’ AI Literacy (SNAIL), alongside self-developed tools. Only one study explicitly defined and measured algorithmic literacy as a distinct construct; in other studies, algorithmic considerations appeared indirectly through recognizing AI presence in systems or evaluating AI generated content. Across studies, competencies aligned mainly with functional and critical dimensions of DHL, particularly awareness, use, evaluation, and ethics, while communicative literacies were infrequently assessed.

**Discussion:**

AI and algorithmic literacy among health workers is underdeveloped, weakly integrated with digital health literacy, and inconsistently measured. Research prioritizes AI literacy using non–health-specific self-report tools and largely overlooks communicative competencies essential to clinical and public health practice. These findings point to the need for clearer conceptual alignment, health-specific measurement, and systems-based approaches to workforce readiness as AI-enabled tools expand across healthcare and public health.

## Introduction

The digital transformation of healthcare and public health has altered professional practice, with algorithmic systems increasingly embedded across clinical and public health workflows, including clinical decision support, surveillance, risk stratification, documentation, and resource allocation (1–3). Prior to the emergence of generative artificial intelligence (AI), algorithms influenced how health information was searched, ranked, filtered, and presented, determining which evidence and guidance were visible to health workers and the public (2–5). The rapid expansion of generative AI and large language models (LLMs) has extended these influences from information retrieval to information synthesis and production (6). AI systems now generate summaries, explanations, recommendations, and draft communications based on algorithmically curated data, often with limited transparency regarding how information is selected, weighted, or framed (2, 3).

Therefore, health workers must navigate not only AI tools, but also the underlying algorithmic processes that structure information exposure and interpretation (7). Health workers are also expected to use AI-enabled systems, interpret, validate, and explain AI-generated and algorithmically mediated health information to patients, communities, and decision-makers in ways that uphold professional judgment, trust, and accountability (7, 8). This positions health workers as intermediaries between digitally mediated systems and those affected by their outputs, with responsibility for interpreting, contextualizing, and, when needed, questioning algorithmic and AI-driven recommendations in clinical care and public health practice (7–9). These expanded responsibilities require new competencies to critically engage with algorithmic outputs and AI-generated content (1, 10). Further, concerns about professional deskilling have emerged, as reliance on algorithmic systems intended to reduce cognitive load may undermine clinical reasoning, public health judgment, and decision-making autonomy over time (11–13). Therefore concerted efforts are needed to address these emergent competency gaps which have become more evident since the COVID-19 pandemic (11, 14).

Digital health literacy has become a central framework for understanding how individuals access, understand, appraise, and apply digitally mediated health information and services for their benefit (15–18). However, existing frameworks have largely focused on health service users, emphasizing information seeking and evaluation (16, 18–21). In contrast, health workers require competencies extending beyond information access to include critical appraisal of AI and algorithmic tools embedded in practice, recognition and mitigation of algorithmic bias, decisions about when to override algorithmic recommendations, and explaining algorithmic processes and outputs to patients, communities, and policymakers in accessible and trustworthy ways (9, 11).

Despite these fundamental role changes, AI and algorithmic literacy among health workers remain poorly defined, particularly in relation to existing digital health literacy frameworks (6). Current approaches show limited conceptual alignment and heterogeneous measurement practices, making it difficult to assess workforce readiness consistently or to support system-level accountability across clinical and public health settings (22). Clarifying how AI and algorithmic literacy are conceptualized and measured is essential to inform workforce development, guide policy, and support evidence-informed practice (11). This is particularly important given evidence that strengthening digital health literacy among health workers remains necessary alongside developing advanced competencies, including AI literacy (14, 23). This study examines how AI and algorithmic literacy are conceptualized and measured among health workers through a digital health literacy lens, and explores implications for adapting existing frameworks to current healthcare and public health practice.

## Methods

### Overview

This study reports findings from a scoping review examining the conceptualization and measurement of AI and algorithmic literacy among health workers. It was conducted using the methodological framework developed by Arksey and O’Malley, with refinements proposed by Levac et al. and guidance from the Joanna Briggs Institute (JBI) (24–26). This framework is particularly well-suited for clarifying complex and emerging concepts, such as the evolving nature of DHL in the context of AI-generated and algorithmically curated health information. Our reporting adheres to the Preferred Reporting Items for Systematic Reviews and Meta-Analyses (PRISMA) for scoping reviews (Appendix 1) (27). A protocol was not registered given the exploratory nature of this scoping review.

### Study eligibility criteria

Eligibility criteria were defined using a modified Population–Concept–Context (PCC) framework, with additional specification of publication language and time frame (Table 1). *Population:* We included literature focusing on health workers, broadly defined to include clinicians, public health practitioners, allied health professionals, and other members of the health workforce involved in healthcare delivery, public health, or health communication. *Concept:* The phenomena of interest were AI literacy and algorithmic literacy in health contexts. Given the emerging nature of these constructs, operational definitions were informed by literature from information and computer science. AI literacy was defined as the capacity to critically evaluate, interact with, and effectively use AI technologies in health contexts, including understanding how AI systems such as LLMs function, recognizing AI as a supportive rather than authoritative source of health information, applying appropriate prompting strategies, critically evaluating outputs, and ethically disclosing AI use where appropriate (6, 28). Algorithmic literacy was defined as the capacity to understand how algorithmic systems influence the visibility, accessibility, and prioritization of health information in digital environments, including the ability to critically assess the implications of algorithmic design and data-driven curation for decision making in health contexts (6, 29). *Context*: We included studies from clinical, public health, and health communication contexts. Literature addressing AI or algorithmic literacy in non-health settings without explicit relevance to health practice or health information environments was excluded. We included peer-reviewed empirical studies, conceptual papers, frameworks, reviews, and relevant grey literature that described, analyzed, or conceptualized AI or algorithmic literacy in health contexts. Only publications in English were included due to resource constraints and to ensure accurate interpretation of conceptual content. Eligible sources were published between January 2020 and May 2025, a period selected based on preliminary searches indicating that AI and algorithmic literacy gained prominence in health-related literature within the past 5 years.

**Table 1:** Eligibility criteria for the scoping review of AI and algorithmic literacy among health workers.

Domain	Inclusion criteria	Exclusion criteria
Population	Literature focusing on health workers, broadly defined to include clinicians, public health practitioners, allied health professionals, and other members of the health workforce involved in healthcare delivery, public health practice, or health communication, including students in training.	Literature focused exclusively on health service users or the public without explicit relevance to health workers or professional practice.
Concept	Studies or reports that describe, analyze, or conceptualize AI literacy or algorithmic literacy in health contexts, including frameworks, models, competencies, domains, or dimensions related to health workers’ engagement with AI-generated or algorithmically curated health information.	Studies focused solely on the technical or engineering design, development, or performance of AI or algorithmic systems without consideration of user-facing literacy, competencies, or skills. Studies addressing general digital literacy without explicit relevance to AI, algorithms, or health contexts.
Context	Studies in healthcare, public health, or health communication contexts, including literature addressing education or training for health workers and professionals.	Studies situated in non-health contexts (e.g., education, finance, entertainment, or general technology use) without explicit relevance to health practice or health information environments.
Types of sources	Peer-reviewed empirical studies (quantitative, qualitative, or mixed methods), conceptual papers, frameworks, reviews, and relevant grey literature (e.g., reports, policy documents, consensus statements, and preprints) that meet inclusion criteria.	Opinion pieces, commentaries, editorials, and viewpoints that do not present conceptual frameworks, analytic insights, or empirical evidence relevant to AI or algorithmic literacy in health contexts.
Language	Publications available in English.	Publications in languages other than English.
Time frame	Publications released between January 2020 and May 2025 (date of final search).	Publications released prior to January 2020.

AI: Artificial intelligence.

### Information sources and search

Our search strategy was informed by concepts related to digital health literacy and eHealth health literacy, alongside emerging descriptions of AI and algorithmic literacy (6, 29–31). The search strategy drew on two complementary approaches. First, we combined terms related to health literacy and health information use (including health literacy, eHealth literacy, health communication, and health information seeking) with terms related to artificial intelligence and algorithmic systems (including artificial intelligence, machine learning, generative AI, large language models, chatbots, and algorithms). Second, we included a set of terms explicitly referring to AI literacy and algorithmic literacy to capture literature that directly conceptualized these constructs (Appendix 2). Searches were conducted on Ovid MEDLINE, Ovid Embase, Scopus, IEEE Xplore, ACM Digital Library, Europe PMC (including preprints), and arXiv. These sources were selected to capture literature spanning healthcare, public health, digital health, and computer science, including relevant grey literature. The final search was conducted on May 5, 2025. Search strategies were refined iteratively through preliminary searches to ensure sensitivity and relevance.

### Selection procedure

**Figure 1:**
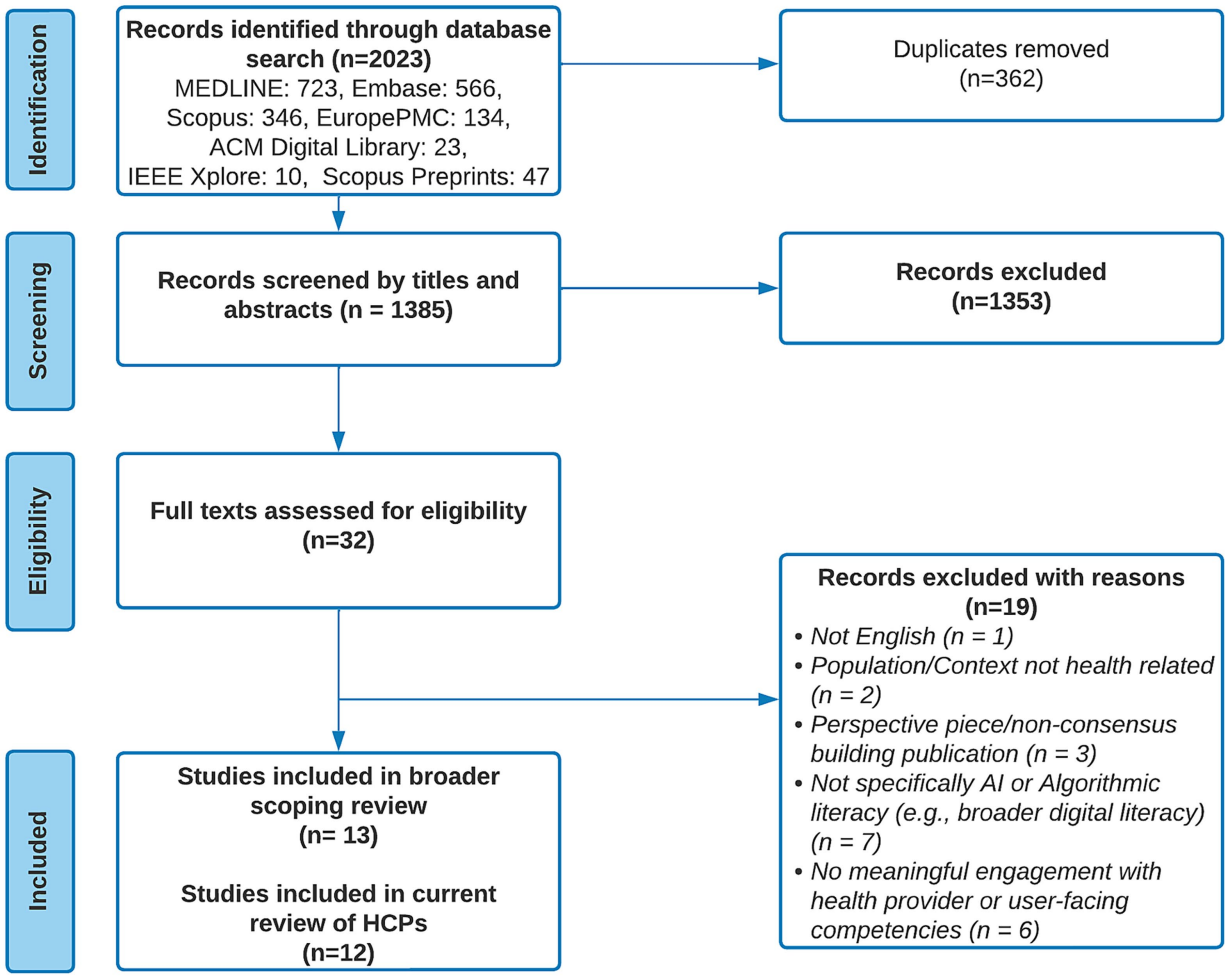
PRISMA flow diagram describing the identification, screening, and selection of evidence sources for this scoping review. ACM, Association for Computing Machinery; HCPs, healthcare providers; IEEE, Institute of Electrical and Electronics Engineers.

All search results were imported into Covidence, a web-based systematic and scoping review management platform, where duplicate records were identified and removed. Study selection was conducted in two stages. First, two reviewers (the first and second authors) independently screened titles and abstracts of all retrieved records against predefined eligibility criteria (Table 1). Second, eligible full texts were independently assessed by the same reviewers using the same criteria. Disagreements at either stage were resolved through discussion and consensus. Interrater agreement was high, with proportionate agreement of 95% (Cohen’s *κ* = 0.33) for title and abstract screening and 84% (Cohen’s κ = 0.66) for full-text screening. The lower kappa value at the title and abstract stage likely reflects the broad and inconsistently applied terminology in this emerging field. Given this variability, inclusion decisions during full-text review prioritized conceptual relevance over strict adherence to specific terms. Studies were included if they substantively engaged with competencies related to interacting with AI-generated or algorithmically curated health information, regardless of the terminology used to describe these constructs. A summary of the study selection process is presented in Figure 1.

### Data charting process

Data from included full-text sources were charted using pretested data extraction forms in Covidence. A structured, theory-informed charting framework was applied to capture both descriptive and conceptual elements relevant to our review objectives (Appendix 1). This theory-informed approach was appropriate for our aim to map how AI and algorithmic literacy are conceptualized and measured in relation to digital health literacy. The charting framework was informed by Ban’s reconceptualization of digital health literacy, which builds on Nutbeam’s three-dimensional model of health literacy, and was supplemented with concepts from Cox’s model of AI literacy (15, 18, 19). Data charting was conducted by one reviewer and independently verified by a second reviewer. Discrepancies were resolved through discussion and consensus. Data items extracted included bibliographic and contextual characteristics (author(s), year of publication, study design, country, population, health setting, and measurement tools used), and conceptual content related to health literacy dimensions (functional (access and basic understanding of digital/AI health content), communicative (interaction with AI systems, including supporting patients to do so), and critical literacies (evaluating the credibility and informed use of health information)) (21). Competencies and dimensions related to AI literacy and algorithmic literacy were charted. The charting framework was refined iteratively during extraction to ensure consistent capture of emerging concepts, consistent with scoping review methodology.

### Analysis and synthesis of results

Both quantitative and qualitative approaches were used to synthesize our review findings. Descriptive statistics were applied to summarize key characteristics of the included studies. For the conceptual synthesis, we conducted a descriptive, theory-informed aggregation of extracted data. Extracted concepts related to AI and algorithmic literacy were first organized within the predefined charting categories, including literacy domains, competencies, and measurement approaches. Indicators used to guide this process included how studies defined or described literacy domains (e.g., functional, communicative, and critical), the types of competencies assessed (e.g., accessing information, interacting with AI systems, evaluating outputs), and how these constructs were operationalized or measured within each study (21). Within these categories, extracted data were reviewed and compared across studies to identify recurring concepts, similarities, and differences in terminology and application. Concepts with shared characteristics were grouped to summarize how AI and algorithmic literacy were described and operationalized across the literature. Theoretical frameworks, including Ban’s reconceptualization of digital health literacy and Cox’s AI literacy model, informed the organization and interpretation of these groupings by providing a lens to situate identified concepts within broader literacy domains and AI-related competencies. These frameworks were used to guide interpretation rather than as formal coding structures (6, 15). The resulting conceptual groupings are presented in this manuscript to illustrate common patterns and areas of divergence in how AI and algorithmic literacy are conceptualized in health contexts. Drawing on these patterns and gaps, we developed a conceptual heuristic AI and algorithmic literacy from a DHL lens, rather than to represent empirically observed relationships between literacies.

## Results

### Description of included studies

Overall, 12 studies published between 2021 and 2025 were included (with 11/12 published in 2024 or later), reflecting the recent emergence of AI and algorithmic literacy in health contexts. Studies were conducted across multiple regions, including Europe, Asia, the Middle East, Africa, and North America (Table 2). Most studies focused on health workers in training (10/12) (32–41), primarily medical students (6/12) (32, 33, 35–37, 41) and nursing students (2/12) (34, 39), but none specifically on public health practitioners. There was limited attention to clinical practice settings and no studies explicitly on public health practice. Most studies (9/12) assessed AI literacy or related competencies, often alongside attitudes toward AI or self-efficacy (32, 34, 35, 37–39, 41–43). Fewer studies examined educational interventions involving generative AI tools (2/12) (38, 39) or conceptualized AI literacy frameworks (2/12) (32, 40). Only one study explicitly examined algorithmic literacy as a distinct construct (36). In contrast, two studies addressed algorithmic issues implicitly, commonly through evaluative tasks such as detecting algorithmic-driven information environments and errors or hallucinations in AI-generated content (35, 39).

**Table 2:** Description of studies included in scoping review of AI and algorithmic literacy among health workers.

Study (Author, year)	Country or region	Population	Study aims relevant to this review	How AI literacy is conceptualized in the study	How algorithmic literacy is conceptualized in the study	How literacy is measured or operationalized	Does the study critique or propose adaptations to digital literacy framework?
Ma et al. preprint (2024) (32)	USA (reviewed globally)	Medical students (through included studies)	To identify existing theoretical proposals, guidelines and practical approaches for teaching AI competencies to medical students	AI literacy presented as four dimensions for the public (know and understand, use and apply, evaluate and create, AI ethics) and proposed medical student AI literacy dimensions (foundational, practical, experimental, ethical)	Not described	No primary measurement instrument. AI literacy competencies were identified through conceptual synthesis in a scoping review of 29 studies, drawing on existing frameworks and curricula. Measurement approaches within included studies were summarized narratively rather than empirically assessed.	Yes. Critiques existing literacy frameworks for limited comprehensiveness, clinical relevance, and personalization. Proposes a medical student AI literacy framework with staged learning objectives
Zhou et al. (2025) (33)	China	General practice trainees	To assess trainees’ ability to recognize AI generated hallucinations in simulated clinical scenarios	AI literacy not defined, only mentioned once as a recommendation to improve	Not described	Performance based assessment task using simulated GPT-4o responses to examination style clinical questions. Participants classified each response as correct, incorrect, or uncertain. Responses were categorized into four cognitive levels (basic knowledge, related professional knowledge, professional knowledge, professional practice). Hallucination detection served as a proxy for evaluative literacy. No standardized literacy scale was used.	No explicit framework critique. Recommends improving AI literacy and critical thinking because trainees struggled to identify errors
Sumengen et al. (2025) (34)	Türkiye	Nursing students	To assess AI literacy levels and attitudes toward AI and examine the relationship between literacy levels and attitudes toward AI	AI literacy treated as a multi-dimensional construct captured by AILS (awareness, usage, evaluation, ethics)	Not described	Artificial Intelligence Literacy Scale (AILS) consisting of 12 items across four subscales: awareness (3 items), usage (3 items), evaluation (3 items), and ethics (3 items), each rated on a 7-point Likert scale. Attitudes toward AI were measured using the General Attitudes Toward Artificial Intelligence Scale (GAAIS)	No framework adaptation described
Mukherjee et al. (2025) (35)	India	First year medical students	To assess AI literacy, self-efficacy, and perceptions of AI among early medical trainees	AI literacy conceptualized as MAILS dimensions (use and apply, know and understand, recognize AI, ethics) plus create AI, self-efficacy, self-management	“Detect AI subscale” partially captures algorithmic awareness through recognition of AI systems	Meta-Artificial Intelligence Literacy Scale (MAILS) comprising 34 items organized into multiple constructs: core AI literacy domains (use and apply AI, know and understand AI, recognize AI systems, AI ethics), plus create AI, AI self-efficacy, and AI self-management. Detect AI subscale assessed recognition of AI systems. Two additional open-ended questions explored perceived advantages and disadvantages of AI.	No explicit DHL framework adaptation described
Kampa and Balzer (2021) (36)	Germany	Medical students	To assess algorithmic literacy using a knowledge-based test focused on algorithms in medicine	Not framed as AI literacy	Algorithmic literacy discussed as linked to data literacy and defined using traditional definitions emphasizing problem solving with algorithmic systems and critical understanding and reflection on algorithmic processes and consequences	Researcher-developed knowledge-based assessment including nine multiple-choice questions using clinical vignettes related to algorithmic systems, each paired with a three-point confidence self-estimation scale (“I do not know,” “I suppose,” “I’m sure”). Three additional Likert-style attitude statements assessed perceptions of algorithmic decision making and digital health education. Authors note the tool assessed cognitive knowledge and excluded pragmatic dimensions.	No DHL framework adaptation described
Laupichler et al. (2024) (37)	Germany	Medical students	To assess AI literacy and attitudes and examine correlates such as age, gender, and training	AI literacy defined using Long and Magerko (Defined as set of competencies that enables individuals to critically evaluate AI technologies; communicate and collaborate effectively with AI; and use AI as a tool online, at home, and in the workplace) and operationalized through SNAIL: technical understanding, critical appraisal, practical application	Not described	Scale for the Assessment of Non-Experts’ AI Literacy (SNAIL) measuring three latent constructs: technical understanding (14 items), critical appraisal (10 items), and practical application (7 items), rated on a 7-point Likert scale. Attitudes toward AI were measured using the Attitudes Toward Artificial Intelligence Scale (ATAI) with two factors: fear (2 items) and acceptance (3 items).	No framework critique or adaptation described
Naamati Schneider (2024) (38)	Israel	Health management students	To explore students’ experiences developing AI related skills through guided ChatGPT use	AI literacy conceptualized in practice as skills to interact with, prompt, critically evaluate, and effectively use AI tools. Also states traditional digital literacy skills are no longer sufficient for AI powered systems	Not described	Qualitative reflective journals completed by students following ChatGPT-mediated guided learning. Data were analyzed thematically. Emergent themes related to AI literacy included prompting competency, evaluation of information credibility, iterative interaction with AI, barriers to use, and dependency on AI systems. No quantitative literacy instrument was used.	Yes, implicitly. Findings suggest shifts in what matters: basic locating and summarizing may become less central as AI improves, while evaluation and critical thinking about AI becomes more important
Kahraman et al. (2025) (42)	Türkiye	Perioperative nurses	To assess AI literacy levels among perioperative nurses	AI literacy conceptualized as AILS domains (awareness, usage, evaluation, ethics)	Not described	Artificial Intelligence Literacy Scale (AILS) consisting of 12 items across four subscales: awareness, usage, evaluation, and ethics (3 items per subscale), rated on a 7-point Likert scale. A researcher developed personal information form collected demographic and AI use characteristics.	No framework critique or adaptation described
Ozcevik Subasi et al. (2024) (43)	Türkiye	Paediatric nurses	To examine AI literacy, anxiety, and attitudes toward AI	AI literacy described using Long and Magerko and operationalized using AILS domains	Not described	AILS (12 items; awareness, usage, evaluation, ethics) used to assess AI literacy. Attitudes toward AI were measured using the GAAIS (20 items; positive and negative attitude subscales). Anxiety related to AI was measured using the Artificial Intelligence Anxiety Scale (AIAS) consisting of 21 items across four subdimensions (learning, job replacement, sociotechnical blindness, AI configuration).	No DHL framework adaptation described
Tseng et al. (2025) (39)	Taiwan	Nursing students	To evaluate the impact of generative AI supported instruction on AI literacy and academic writing	AI literacy conceptualized using MAILS dimensions. MAILS includes AI self-management which relates to influence and emotion regulation	“Detect AI” partially captures algorithmic awareness through recognition of AI systems	Meta-AI Literacy Scale (MAILS) assessing AI literacy across multiple dimensions: AI literacy core facets (use and apply AI, understand AI, recognize AI systems, AI ethics), create AI, AI self-efficacy, and AI self-management. Literacy outcomes were complemented by rubric-scored academic case reports assessed by three instructors with high inter-rater agreement.	No explicit DHL framework adaptation described
Zary (2025) preprint (40)	UAE	Clinicians, educators, researchers, students, administrators (through included studies)	To develop a comprehensive AI literacy framework applicable across healthcare roles	AI literacy conceptualized as a structured framework with progression from foundational literacy to leadership capability, integrating technical understanding, practical application, critical evaluation, ethical considerations, and data literacy	Not described	No empirical measurement tool. AI literacy competencies were operationalized through framework development, synthesizing existing literature to define progressive competency levels across technical understanding, practical application, critical evaluation, ethical considerations, and data literacy.	Yes. Critiques existing frameworks for limited role coverage and limited progression pathways. Proposes ALiF with staged progression, role specificity, and practical assessment strategy
Subaveerapandiyan et al. (2024) (41)	Zambia	Medical students	To assess AI literacy, awareness, perceived benefits, and concerns regarding AI in healthcare	AI literacy defined as multifaceted and including digital literacy, computational thinking, and comprehension of an intelligent society. In healthcare, ability to engage with AI tools for multiple purposes	Not described, although the aim includes concerns about algorithm training	Self-developed closed-ended questionnaire, pilot tested and reviewed by content experts. Items assessed familiarity with AI concepts, perceived ability to evaluate AI generated information, understanding of limitations and ethical considerations, perceived benefits and risks, and use of AI tools such as chatbots. Specific item counts and subscale structure were not reported in the extract.	No explicit DHL framework adaptation described

AI, artificial intelligence; AILS, Artificial Intelligence Literacy Scale; AIAS, Artificial Intelligence Anxiety Scale; ALiF, AI Literacy Framework; ATAI, Attitudes Toward Artificial Intelligence scale; DHL, digital health literacy; GAAIS, General Attitudes Toward Artificial Intelligence Scale; GPT, generative pretrained transformer; MAILS, Meta Artificial Intelligence Literacy Scale; SNAIL, Scale for the Assessment of Non-Experts’ AI Literacy.

### AI and algorithmic literacy dimensions among health workers and their alignment with digital health literacy

Across the included studies, explicit, theory grounded definitions of AI literacy were uncommon, and links to established DHL constructs were often implied rather than clearly articulated (34, 35, 39, 41, 42). Only a small subset of studies provided an explicit conceptual definition, most often drawing on Long and Magerko’s framework, which conceptualizes AI literacy as a set of competencies enabling individuals to evaluate AI technologies, communicate and collaborate with AI systems, and use AI tools across professional contexts (37, 40, 43). When applied in healthcare settings, however, these definitions were operationalized primarily through functional and critical dimensions of digital health literacy, emphasizing understanding, ethical use, and appraisal of AI outputs in clinical decision making (32, 35, 38, 40–43). One additional study conceptualized AI literacy as the capacity to understand and interact with AI technologies, highlighting its multifaceted nature (41). While not explicitly framed through a DHL lens, this definition implicitly emphasized functional literacy, particularly basic understanding and interaction with AI systems, with more limited attention to communicative literacy which was described in relation to integrating AI systems in workflows and collaborating effectively with them rather than competencies related to explaining AI-mediated information, negotiating uncertainty, or supporting patient and community decision making. In contrast, one study referenced AI literacy only briefly without definition or conceptual framing, underscoring ongoing ambiguity in how the construct is positioned relative to existing health literacy frameworks (33).

Algorithmic literacy was explicitly defined and examined in only one study, where it was framed primarily as cognitive understanding of algorithmic processes, limitations, and risks in medicine, with the authors noting the exclusion of pragmatic and applied dimensions (36). Across the remaining studies, algorithmic considerations were addressed implicitly rather than conceptualized as a distinct literacy domain, most often through recognition of AI systems or evaluation of AI-generated outputs, such as detecting hallucinations or assessing ethical risks (33–35, 39, 42, 43). The emphasis was largely on functional and critical literacies (34, 35, 37, 39, 40, 42, 43). In contrast, communicative literacy was largely absent from both conceptual frameworks and measurement instruments.

### Measurement of AI and algorithmic literacy among health workers and implications for digital health literacy frameworks

AI literacy was most frequently measured using quantitative self-reported instruments, with limited use of performance-based or qualitative approaches. Seven studies operationalized AI literacy through measurement tools without providing explicit definitions, allowing the construct to be inferred from the domains assessed (33–35, 39, 41–43). The Artificial Intelligence Literacy Scale (AILS) was the most used instrument (3/12 studies) (34, 42, 43). AILS consists of 12 items distributed evenly across four subscales: awareness (three items), usage (three items), evaluation (three items), and ethics (three items), each rated on a 7-point Likert scale (44). AILS was used primarily among nursing students and practicing nurses. The Meta-Artificial Intelligence Literacy Scale (MAILS) was used in two studies, both in health professions education. MAILS includes 34 items spanning four core AI literacy domains (use and apply AI, know and understand AI, recognize AI systems, AI ethics) alongside additional constructs (create AI, AI self-efficacy, and AI self-management) (35, 39, 45). In both studies, MAILS was complemented by open-ended questions or performance-based academic assessments, indicating broader operationalization of AI-related competencies. The Scale for the Assessment of Non-Experts’ AI Literacy (SNAIL) was used in one study (37). SNAIL includes 31 items assessing three constructs: technical understanding (14 items), critical appraisal (10 items), and practical application (7 items), measured on a 7-point Likert scale (46). This instrument was validated among medical students and represents the most detailed assessment of AI-related competencies within a health-related population. Three studies used self-developed questionnaires, though reporting on item counts and construct structure varied (33, 36, 41). These tools generally assessed familiarity with AI concepts, perceived ability to evaluate AI-generated outputs, ethical considerations, and perceived benefits and risks of AI use. In one study, item counts and subscale structures were not reported (41). Only one study employed a performance-based assessment, using simulated AI-generated clinical responses to evaluate trainees’ ability to identify hallucinations (33). This task classified responses across four cognitive levels and did not rely on a standardized literacy scale. One qualitative study used reflective journals to examine students’ interactions with generative AI tools (38). While no itemized instrument was used, the analysis identified domains related to prompting, credibility assessment, iterative interaction, and dependency.

Algorithmic literacy was explicitly defined and measured in only one study (36). This assessment consisted of nine multiple-choice knowledge questions based on clinical vignettes, each paired with a three-point confidence rating, alongside three Likert-style attitude statements. The instrument focused on cognitive understanding of algorithmic systems. Overall, measurement varied substantially in item counts, constructs assessed, and methodological approaches, with most tools emphasizing self-reported awareness, use, evaluation, and ethical considerations of AI systems. Explicit assessment of algorithmic literacy and practice-based competencies was uncommon.

## Discussion

### Summary of results

This scoping review identified substantial variability and limited conceptual clarity in how AI and algorithmic literacy are defined, operationalized, and measured among health workers. The evidence base is recent and concentrated largely in health professions education, particularly among medical and nursing students, with limited attention to routine clinical or public health practice. AI literacy was examined more frequently than algorithmic literacy and was most often inferred through measurement tools rather than explicitly theorized. When definitions were provided, AI literacy was typically framed as a multidimensional construct encompassing understanding, use, evaluation, and ethical engagement with AI systems, drawing primarily on frameworks developed outside healthcare and not well linked to DHL. Algorithmic literacy was rarely directly measured and was usually addressed indirectly through evaluative tasks related to AI-generated content. Measurement approaches were dominated by self-reported instruments emphasizing awareness, use, evaluation, and ethics, aligning most closely with functional and critical dimensions of DHL, while communicative competencies and practice-based assessment were infrequently examined.

### Comparison with the literature and unique contributions

This study contributes to a rapidly evolving body of literature on AI and algorithmic literacy in workforce contexts, highlighting the limited adaptation of curricula to ongoing digital transformation (14, 47, 48). Consistent with prior research on digital competencies in the health workforce, existing work remains largely focused on clinical and educational settings, with comparatively limited attention to public health practice (23, 49). Similar to earlier work, we observed a strong focus on functional and critical literacies related to accessing, appraising, and ethically using digital health information (7, 8, 50). This emphasis was reflected in the predominance of competencies linked to responsible AI use within governance, regulatory, and legal frameworks (9, 50, 51). Consistent with other reviews, the studies included here relied heavily on influential AI literacy frameworks developed outside healthcare and public health (52–54). Measurement approaches similarly mirrored earlier DHL and eHealth competency research, relying on self-reported instruments that emphasize perceived skills over observed practice (48, 53, 55). These patterns suggest that while existing frameworks and measures have provided a useful foundation, they may insufficiently capture the competencies required for AI-enabled clinical and public health practice, particularly those related to population-level decision-making, risk communication, and equity.

This review also highlights persistent gaps in the literature. Although contemporary AI literacy frameworks increasingly acknowledge higher-order competencies such as collaboration with AI systems and reflective judgment, these elements are inconsistently operationalized in health workforce research (11). Communicative competencies central to health literacy frameworks, including explaining AI-mediated information, contextualizing uncertainty, and supporting shared decision-making, remain largely absent from empirical studies and measures (11, 48). In addition, algorithmic literacy, which has been more extensively theorized in communication, media, and information sciences as a determinant of information exposure, bias, and trust, is minimally integrated into health workforce research (6). A recent algorithmic literacy framework (ALF) proposed for the global health workforce suggests that algorithmic literacy enables people to recognize the impact of algorithmically driven systems and to strategically engage with them, including understanding how curated content may prioritize or omit information (47). While this represents an important development, it remains a relatively isolated contribution. This highlights a gap between conceptual advances in understanding algorithmically mediated information environments and the capacities expected of the health workforce to navigate these systems and support communities in doing the same (11, 56). While algorithms have long influenced health information exposure, their normalization as infrastructure limit explicit literacy development. Recent interest in generative AI has made these dynamics more visible, yet workforce competencies remain underdeveloped.

### Implications for policy, practice and research

Our findings reveal under-developed conceptualizations and fragmented measurement of algorithmic and AI literacy, limiting clarity about workforce competencies in AI-enabled practice. This indicates that workforce readiness is not being systematically addressed alongside technological change, underscoring the need to align workforce capacity with the design, governance, and deployment of AI-enabled healthcare and public health systems (9, 14, 57). Current approaches to AI literacy emphasize precautionary and risk-oriented framings, particularly ethics, governance, and error detection (8, 50, 51). While these dimensions are essential, their dominance reflects the influence of structured training contexts and risks placing disproportionate responsibility on individual health workers to manage system-level complexity. As AI-enabled tools increasingly impact clinical workflows, public health practice, and health information environments already used by the public, health workers are positioned as intermediaries between algorithmically mediated systems and communities (11, 58). Our findings emphasize the need for not only technical understanding, but also communicative and interpretive competencies that support trust, accountability, and appropriate use, which must be enabled by supportive organizational, regulatory, and technological infrastructures (10, 11, 14). For example, health workers may be trained to contextualize AI-generated risk information, communicate uncertainty, and address misconceptions arising from algorithmically mediated health content during clinical encounters or public health communication.

The minimal attention to algorithmic literacy is notable given extensive evidence from outside healthcare demonstrating that algorithmic systems significantly influence information exposure and perpetuate existing inequities (4, 6). Without literacies that support recognition of these dynamics, health workers may be limited in their ability to identify, question, or mitigate algorithmic influences on care delivery and population-level decision making (4, 5). However, our findings suggest that improving AI and algorithmic literacy cannot be treated as an individual responsibility alone, but must occur alongside investments in transparent system design, governance mechanisms, and standards that support explainability and equity (10). For education, our findings underscore the need to extend beyond undergraduate and postgraduate training to include continuing professional development, and develop health-specific, practice-relevant measurement tools that capture how these literacies operate in real-world settings (14, 59). Such efforts are contingent on a nascent research agenda. Conceptually, clearer theorization is needed to define the relationship between algorithmic literacy, AI literacy, and digital health literacy. Methodologically, validated healthcare-specific measures of AI and algorithmic literacy are required. Empirically, studies must move beyond training contexts to examine how these literacies function in routine clinical and public health practice and how they relate to trust, equity, and system accountability.

### Toward an integrated digital health literacy framework for AI-enabled healthcare and public health

**Figure 2:**
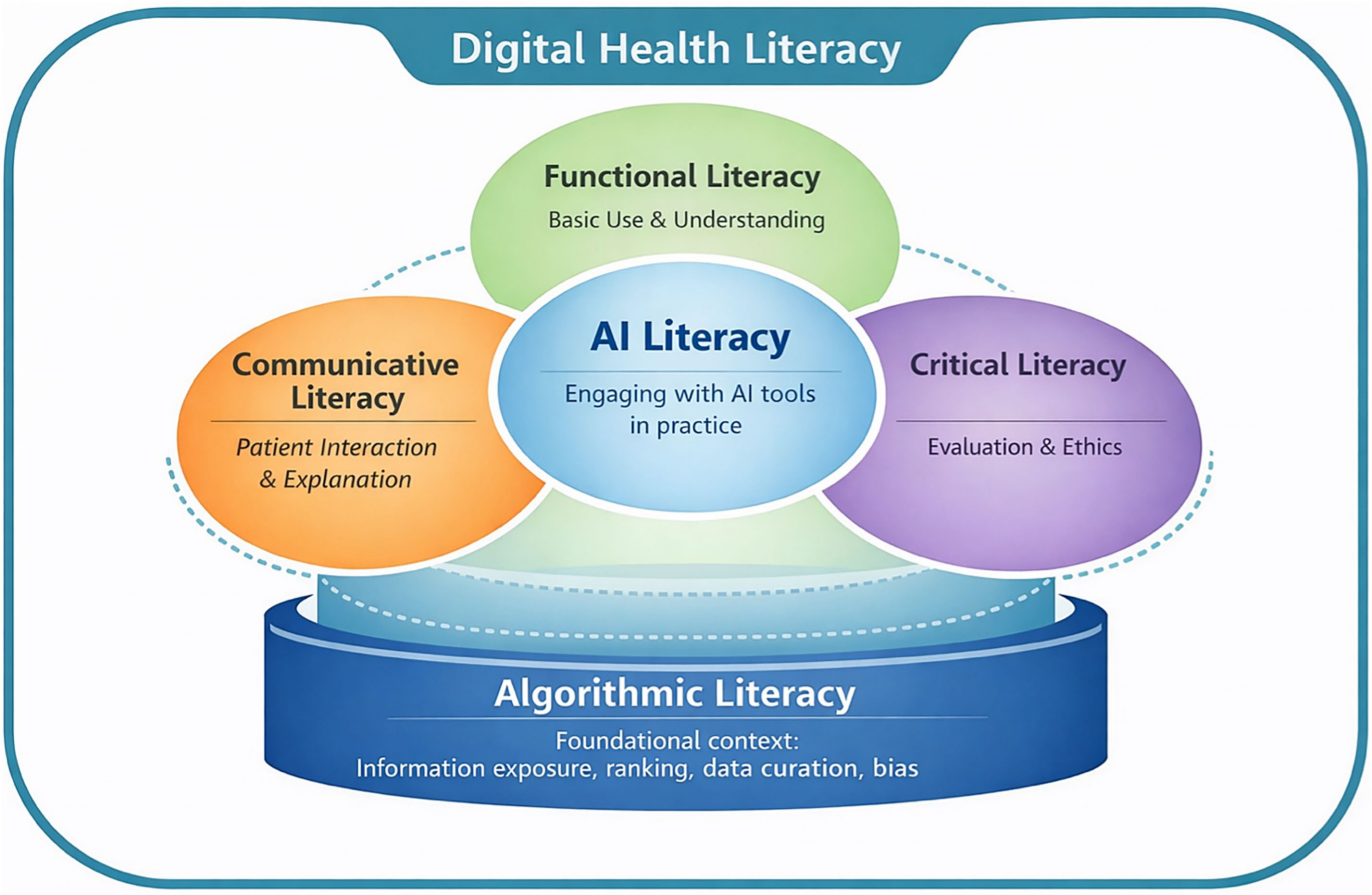
Conceptual heuristic illustrating how algorithmic literacy informs functional and critical dimensions of digital health literacy among health workers, and how AI literacy is enacted through functional, critical, and communicative competencies in health practice.

Drawing on patterns identified in this review, we propose a conceptual synthesis clarifying the relationships between algorithmic literacy, AI literacy, and DHL among health workers (Figure 2). This synthesis is offered as a heuristic rather than a validated framework, intended to support clearer conceptualization and future measurement development. Across included studies, AI-related literacies were commonly assessed without explicit attention to how underlying algorithms shape outputs, information exposure, and decision pathways. In this heuristic, we position algorithmic literacy as an underpinning layer, referring to the capacity to recognize and critically understand how data sources, model design, and algorithmic curation shape information environments, access, and potential bias (9, 14, 56). AI literacy builds on this foundation and refers to the ability to engage with AI systems in practice, including interpreting, evaluating, and appropriately using outputs. Without an algorithmic literacy foundation, AI literacy risks overemphasizing tool interaction while overlooking structural influences on bias, accountability, and decision-making (9). We do not propose this ordering as a settled hierarchy, but as a useful conceptual distinction for clarifying measurement and workforce expectations in AI-mediated health information environments. Situating both within a DHL framework highlights that effective and safe use of AI in healthcare and public health requires not only established DHLs, but also the ability to access, understand, appraise, and apply digital health information while understanding the systems and mechanisms that influence its generation and access (16, 18, 21). Within this framing, algorithmic and AI literacies extend these DHLs by addressing how information is generated, curated, and interpreted in AI-mediated environments. This is particularly important as patients and communities increasingly use AI and algorithmically curated platforms including social media to inform health decisions (58).

### Strengths and limitations

This scoping review addresses a timely and policy-relevant gap by examining how AI and algorithmic literacy are conceptualized and measured among health workers through a digital health literacy lens. Strengths include a multidisciplinary, theory-informed approach integrating health literacy, digital health, communication, and AI literacy frameworks, alongside transparent methods consistent with the Arksey and O’Malley framework and PRISMA-ScR reporting (27). Searches across health, public health, and computing databases enabled comprehensive mapping of this rapidly evolving field. The evidence base, however, is heavily concentrated in health professions education, particularly among medical and nursing students, with limited representation of practicing clinicians or public health professionals. Therefore, findings primarily reflect structured training contexts rather than routine care. This limitation affects our understanding of how AI and algorithmic literacies are enacted and sustained in real-world, AI-embedded contexts and patient interactions. As the field is rapidly evolving, studies published after our search may not have been captured. Further research is needed to advance this area. As a scoping review, this study did not assess study quality and maps the breadth rather than the strength of evidence. Conceptual inconsistency across studies limits comparability but also represents a central finding. Rapid advances in AI, restriction to English-language publications, and exclusion of commentaries may have further limited capture of emerging practice-oriented perspectives. These limitations highlight the need for research and policy efforts that extend beyond undergraduate and postgraduate training to include continuing medical education and professional development as AI-enabled systems continue to evolve.

## Conclusion

This scoping review shows that AI and algorithmic literacy among health workers is underdeveloped, weakly integrated with DHL, and inconsistently measured. Across studies, measurement emphasizes functional and critical literacies, with limited attention to communicative and interpretive competencies essential for patient-centered care, public communication, and population-level decision-making. These findings support three recommendations. First, clearer conceptual clarity is needed between AI and algorithmic literacy to define workforce expectations and support accountability. Second, health-specific, practice-sensitive measurement tools are required to assess these literacies in real-world clinical and public health settings. Third, strengthening AI and algorithmic literacy should be approached as a system-level responsibility. This is needed to align workforce development with transparent, well-governed, and trust-enabling AI infrastructures, rather than placing responsibility primarily on individual health workers. The heuristic proposed in this review offers a starting point for situating AI and algorithmic literacy within a digital health literacy framework, supporting future research, measurement development, and policy discussions aimed at preparing the health workforce for AI-enabled healthcare and public health systems.

## Data Availability

The original contributions presented in the study are included in the article/Supplementary material, further inquiries can be directed to the corresponding author/s.

## Supplementary material

SUPPLEMTNARY TABLE 1 Preferred Reporting Items for Systematic reviews and Meta-Analyses extension for Scoping Reviews (PRISMA-ScR) Checklist.

SUPPLEMTNARY TABLE 2 Search strategy across multiple databases.
