# Supplementary material for: AI and algorithmic literacy among health workers: a scoping review through a digital health literacy lens
Source: Front Public Health. 2026 Apr 23;14:1802392. doi: 10.3389/fpubh.2026.1802392 (PMC13149243; doi:10.3389/fpubh.2026.1802392)
Supplement: SUPPLEMTNARY TABLE 2 — Search strategy across multiple databases. [file Table_2.DOCX]

## *Appendix 2: Search strategy*

**Database:** Ovid MEDLINE(R) ALL <1946 to May 5, 2025>

| **#** | **Query** | **Results from May 5, 2025** |
| --- | --- | --- |
| 1 | exp Health Literacy/ | 11742 |
| 2 | (health literacy or ehealth literacy).tw,kf. | 19726 |
| 3 | (health communication or health information seeking).tw,kf. | 7615 |
| 4 | 1 or 2 or 3 | 29582 |
| 5 | exp Artificial Intelligence/ | 236378 |
| 6 | (artificial intelligence or AI or machine learning or ChatGPT or GPT* or chatbot* or Gemini or algorithm* or genAI or LLM* or large language model*).tw,kf. | 625417 |
| 7 | 5 or 6 | 740990 |
| 8 | 4 and 7 | 745 |
| 9 | (algorithm* literacy or AI literacy or artificial intelligence literacy).tw,kf. | 120 |
| 10 | 8 or 9 | 861 |
| 11 | limit 10 to (english language and yr="2020 -Current") | 723 |

**Database:** OVID Embase

| **#** | **Query** | **Results from May 5, 2025** |
| --- | --- | --- |
| 1 | exp Health Literacy/ | 25650 |
| 2 | (health literacy or ehealth literacy).tw,kf. | 24424 |
| 3 | (health communication or health information seeking).tw,kf. | 7863 |
| 4 | 1 or 2 or 3 | 39051 |
| 5 | exp Artificial Intelligence/ | 137929 |
| 6 | (artificial intelligence or AI or machine learning or ChatGPT or GPT* or chatbot* or Gemini or algorithm* or genAI or LLM* or large language model*).tw,kf. | 767628 |
| 7 | 5 or 6 | 800390 |
| 8 | 4 and 7 | 937 |
| 9 | (algorithm* literacy or AI literacy or artificial intelligence literacy).tw,kf. | 81 |
| 10 | 8 or 9 | 1016 |
| 11 | limit 10 to (english language and yr="2020 -Current") | 848 |
| 12 | limit 11 to (embase or "preprints (unpublished, non-peer reviewed)") | 566 |

**Database:** SCOPUS

| **#** | **Query** | **Results from May 5, 2025** |
| --- | --- | --- |
| 1 | ( TITLE-ABS-KEY ( "health literacy" OR "ehealth literacy" OR "health communication" OR "health information seeking" ) AND TITLE-ABS-KEY ( "artificial intelligence" OR ai OR "machine learning" OR chatgpt OR gpt* OR chatbot* OR gemini OR algorithm* OR genai OR llm* OR "large language model*" ) ) OR ( TITLE-ABS-KEY ( ( "algorithm* literacy" OR "AI literacy" OR "artificial intelligence literacy" ) AND ( health* OR medic* ) ) ) AND PUBYEAR > 2019 AND PUBYEAR < 2026 AND NOT INDEX ( medline ) AND NOT INDEX ( embase ) AND ( EXCLUDE ( DOCTYPE , "ch" ) ) | 346 |

^*^Includes papers from ACM and IEEE conferences (full papers not just abstracts); also includes 54 preprints from arXiv and SSRN.

**Database:** ACM

| **#** | **Query** | **Results from May 5, 2025** |
| --- | --- | --- |
| 1 | [[All: "artificial intelligence"] OR [All: ai] OR [All: "machine learning"] OR [All: chatgpt] OR [All: gpt*] OR [All: chatbot*] OR [All: gemini] OR [All: algorithm*] OR [All: genai] OR [All: llm*] OR [All: "large language model*"]] AND [[Title: "health literacy"] OR [Title: "ehealth literacy"] OR [Title: "health communication"] OR [Title: "health information seeking"]] AND [E-Publication Date: (01/01/2020 TO 31/12/2025)] | 15 |
| 2 | [[Title: "ai literacy"] OR [Title: "algorithm* literacy"] OR [Title: "artificial intelligence literacy"]] AND [All: health or healthcare or medic*] AND [E-Publication Date: (01/01/2020 TO 31/12/2025)] | 8 |

**Database:** IEEE Xplore

| **#** | **Query** | **Results from May 5, 2025** |
| --- | --- | --- |
| 1 | ("Document Title":"health literacy" OR "Document Title":"ehealth literacy" OR "Document Title":"health communication" OR "Document Title":"health information seeking") AND ("All Metadata":"artificial intelligence" OR "All Metadata":ai OR "All Metadata":"machine learning" OR "All Metadata":chatgpt OR "All Metadata":gpt* OR "All Metadata":chatbot* OR "All Metadata":gemini OR "All Metadata":algorithm* OR "All Metadata":genai OR "All Metadata":llm* OR "All Metadata":"large language model*") | 11 |
| 2 | ("Document Title":"AI literacy" OR "Document Title":"algorithm* literacy" OR "Document Title":"artificial intelligence literacy") AND ("All Metadata":health OR "All Metadata":healthcare OR "All Metadata":medic*) | 1 result, from IT Professional magazine (did not export to Covidence) |

**Database:** Europe PMC

| **#** | **Query** | **Results from May 5, 2025** |
| --- | --- | --- |
| 1 | (TITLE_ABS:(AI OR "artificial intelligence" OR "machine learning" OR chatgpt OR gpt* OR chatbot* OR gemini OR algorithm* OR genai OR llm* OR "large language model*") AND TITLE_ABS:("health literacy" OR "ehealth literacy" OR "health communication" OR "health information seeking" )) AND (SRC:PPR) | 125 |
| 2 | (TITLE_ABS:("AI literacy" OR "algorithmic literacy" OR "artificial intelligence literacy") AND TITLE_ABS:(health* OR medic*)) AND (SRC:PPR) | 9 |
